# Mindfulness in psychiatry – where are we now?

**DOI:** 10.1192/pb.bp.115.052993

**Published:** 2016-12

**Authors:** Paramabandhu Groves

**Affiliations:** 1 Camden and Islington NHS Foundation Trust, London

## Abstract

Mindfulness is an increasingly popular therapeutic approach. Mindfulness-based interventions have been tried out in a wide range of mental disorders, with the strongest evidence for use in depression and anxiety. Mindfulness operates by changing the person's relationship with unhelpful thoughts and emotions. The need for home practice is both a strength and a weakness. Some find home practice too demanding and a barrier to effective utilisation of mindfulness. Others discover a set of practical tools that, once learnt, can be applied to ongoing life difficulties; in this way mindfulness may have a place in promoting recovery beyond the acute treatment of a disorder. Additionally, mindfulness may be beneficial for clinicians to promote well-being and enhance the therapeutic relationship.

Over the past 15 years there has been a surge of interest in mindfulness as a therapeutic modality. Mindfulness is being applied to an increasingly diverse range of conditions and the number of academic publications on the subject has grown exponentially. In the UK the interest is so great that an all-party group was formed to look at the potential benefits of mindfulness. Their recently produced report (*Mindful Nation UK*)^1^ made recommendations for using mindfulness in health, education, criminal justice and the workplace.

## What is mindfulness?

A popular working definition of mindfulness coined by Jon Kabat-Zinn is learning to pay attention moment by moment, intentionally, and with curiosity and compassion.^2^ Any object, internal or external, can be the focus of mindful attention; however, in mindfulness training for psychiatric disorders the chief emphasis is on deliberately attending to internal experience, such as body sensations, thoughts and emotions. There is a stress on noticing direct experience, rather than getting caught up in the stories about the experience. Importantly, the quality of attention is essentially non-judgemental, emphasising interest and a sense of kindness to whatever experience arises. Thus a core feature of mindfulness is about how we pay attention to experience, rather than the content of experience *per se*.

## A brief history of mindfulness in the West

Mindfulness has been practised for over 2 millennia, in particular in the Buddhist tradition. In the late 1970s Kabat-Zinn set up a clinic in Massachusetts, USA, inviting physicians to refer people with chronic pain or stress. He delivered an 8-week course in mindfulness, and showed that it benefited people with chronic pain, also at a 4-year follow-up.^3,4^ The course came to be called mindfulness-based stress reduction (MBSR) and was found to be helpful for a variety of conditions including anxiety,^5^ psoriasis,^6^ fibromyalgia^7^ and enhancing immune function.^8^

Despite its popularity in North America, mindfulness for health problems did not catch on greatly in the UK until the work of Zindel Segal, Mark Williams and John Teasdale.^9^ In 1992 they were tasked with developing a maintenance form of cognitive therapy that could prevent further episodes of depression in those currently recovered, using a group format for cost-effectiveness. Initially they intended to inject a little mindfulness into a cognitive-behavioural therapy course, but ended up with a mindfulness course with some elements added from cognitive therapy. Their course, mindfulness-based cognitive therapy (MBCT), drew heavily on Kabat-Zinn's MBSR. A three-centre randomised controlled trial showed that MBCT halved the relapse rate in those with three or more episodes of depression.

The publication of their work engendered a burgeoning interest in the application of mindfulness as a therapeutic approach, producing a whole family of mindfulness-based interventions (MBIs). As well as being used as a stand-alone treatment, mindfulness is also a component of other treatment modalities such as dialectical behaviour therapy (DBT) and acceptance and commitment therapy (ACT).

## How does mindfulness work?

The simple version – useful for explaining mindfulness to course participants – is the ABC of MBIs. ‘A’ stands for developing awareness. This is the foundation, with a particular emphasis on cultivating body awareness. Usually the first main formal mindfulness practice that is introduced is the body scan, in which one is led through paying attention systematically to different parts of the body. Usually within moments of starting the practice, the mind gets caught up in thinking about something else. The basic instruction is to notice when the mind wanders off and then return to the body sensations. Noticing where the mind has gone off to, one discovers in detail the habitual patterns of the mind. Returning back to the body teaches how to step out of being caught up in mental distractions.

‘B’ is for being with experience. Building on the foundation of body awareness, once a person has gained some familiarity with noting the mind going off and returning back to the object of meditation (such as the body sensations), the next step is to learn to turn towards difficult experience with an attitude of acceptance. This counters typical reactions of pushing away unwanted experience. Finally, ‘C’ is for making wise choices. Having learnt to stay with persistent or painful experiences, one is in a better position to judge what the most helpful thing to do next is.

Being with difficult thoughts or emotions is the crux of MBIs, but it can feel counterintuitive. Usually, when difficult experiences arise they are avoided (e.g. by the use of substances in addiction) or one is sucked into them in an unhelpful way (such as with rumination in depression). MBIs, through turning towards unwanted experience, steer away from these twin responses of ‘blocking’ or ‘drowning’. In many disorders there is an overuse of a conceptual narrative focus; for example, in recurrent depression, getting caught up in thoughts such as ‘Why do I feel so bad?’, ‘What is wrong with me?’ or in social phobia, ‘What if I can't get my words out? People can see that I am red and sweaty’.

A study using functional magnetic resonance imaging (fMRI) suggests that mindfulness training leads to a move away from narrative focus to experiential focus (attention to direct sensory experience).^10^ Training produces a decoupling of the medial prefrontal cortex (associated with narrative focus) from the insula (viscero-somatic signals), and an increased connection between the insula and the lateral prefrontal cortex (associated with experiential focus). That is, thoughts, feelings and body sensations are seen less as good or bad, and more as transient mental events.

## What is the evidence for MBIs?

The original work with MBCT has been extended to suggest promising evidence for use in treatment-resistant depression,^11,12^ in people with current symptoms of anxiety or depression,^13^ including those currently depressed and suicidal,^14^ and in improving residual symptoms regardless of the number of previous episodes of depression.^15^ A recent trial found similar relapse rates with those who used MBCT to help taper off and discontinue antidepressant medication (44% relapsed), and those remaining on antidepressant medication without using MBCT (47% relapse rate).^16^ This study indicated that those with a history of abuse did particularly well with MBCT, suggesting that people most at risk of relapse might benefit most from MBCT. In bipolar disorder MBCT does not appear to prevent relapse, but may reduce anxiety and depressive symptoms between episodes.^17,18^

Mindfulness may help to prevent relapse into addiction by interrupting the automaticity of substance use in response to triggers and by reducing unhelpful attention biases and memory responses to triggers.^19^ A review of seven randomised controlled trials showed that substance use improved in five studies and was similar to controls in two studies,^20^ with benefits also found in subsequent studies.^21,22^ There were improvements in some other outcomes such as overall psychological and social adjustment. Patient satisfaction was high and between half and 80% continued to practise mindfulness after the programme had finished.

In the past meditation was thought to be contra-indicated in people with psychosis. However, recent pilot studies have shown that mindfulness may be safe. In this population, it is recommended that practices are shorter, guidance is more frequent, and explicit reference is made to psychotic phenomena.^23^ Mindfulness may promote acceptance of psychotic experiences and increase the capacity to disengage from them.

Other areas with initial evidence for the usefulness of mindfulness include eating disorders,^24^ obsessive-compulsive disorder,^25^ hypochondriasis,^26^ somatisation disorders (especially irritable bowel syndrome),^27^ autism-spectrum disorders (in adults)^28^ and attention-deficit hyperactivity disorder.^29^

A meta-analysis by Khoury *et al*^30^ concluded that MBIs are an effective treatment for a variety of psychological problems, and especially for reducing anxiety, depression and stress. They included 209 studies that consisted of pre-post comparisons, waiting list controls and comparisons with other active treatments. Not surprisingly, the strongest evidence was for pre-post comparisons and against waiting list controls. MBIs were more effective than some psychological treatments, although there was no difference in effectiveness when compared with traditional cognitive–behavioural therapy, behaviour therapies or pharmacological treatments.

An overview of reviews and meta-analyses by Gotink *et al*^31^ suggested that, compared with waiting list controls and other active treatments, MBIs improved depressive symptoms, anxiety, stress, quality of life and physical functioning.

## What is the place of mindfulness now?

Mindfulness does not appear to be more effective than other established treatments. However, where there is an evidence base, it offers an alternative treatment approach, which may either be a sufficient treatment in its own right or may complement other strategies. Thus, mindfulness may increase the range of treatment choices for patients. Given the potential usefulness of mindfulness in depression and anxiety as well as in addiction, it has been suggested that mindfulness may also be a helpful approach for patients with dual diagnosis.^32^

The plethora of popular books on mindfulness suggests that it may have a wide appeal. One of the main factors that can put patients off is the home practice, which some find too demanding. Typical courses in MBIs are characterised by daily home practice for up to an hour, although there is a trend towards shortening the practices. Home practice appears to be linked to outcome, but the minimum effective dose is not known. The corollary is that those who are able to practise at home report coming away from a course with a practical toolkit that they can continue to use after the course has finished.

A possible advantage of mindfulness is as a life skill that goes beyond the immediate treatment of a particular disorder. Mindfulness training may therefore dovetail well with the emphasis in treatment services on recovery. Some patients may perceive mindfulness as a spiritual approach to mental health problems, without needing to be affiliated to any particular religious group.

Finally, practising mindfulness may be of benefit for clinicians themselves. The *Mindful Nation UK* report^1^ recommends developing mindfulness programmes for public sector staff to combat stress and improve organisational effectiveness. A number of studies have been conducted among medical students and other health professionals. As well as improving well-being,^33^ reducing mood disturbance and helping to deal with stress,^34^ mindfulness training appears to increase empathy levels.^35^ Thus, mindfulness may have the potential to enhance the therapeutic relationship and so benefit clinical outcomes.
